# Using qualitative comparative analysis to understand the conditions that produce successful PrEP implementation in family planning clinics

**DOI:** 10.1186/s43058-023-00450-2

**Published:** 2023-06-09

**Authors:** Kaitlin N. Piper, Katherine M. Anderson, Caroline W. Kokubun, Anandi N. Sheth, Jessica Sales

**Affiliations:** 1 Rollins School of Public Health, Emory University, Atlanta, GA USA; 2 Department of Medicine, Division of Infectious Diseases, Emory University School of Medicine, Atlanta, GA USA

**Keywords:** Pre-exposure prophylaxis (PrEP), HIV, Family planning, Configurational comparative methods, Implementation

## Abstract

**Background:**

Title X-funded family planning clinics have been identified as optimal sites for delivery of pre-exposure prophylaxis (PrEP) for HIV prevention to U.S. women. However, PrEP has not been widely integrated into family planning services, especially in the Southern U.S., and data suggest there may be significant implementation challenges in this setting.

**Methods:**

To understand contextual factors that are key to successful PrEP implementation in family planning clinics, we conducted in-depth qualitative interviews with key informants from 38 family planning clinics (11 clinics prescribed PrEP and 27 did not). Interviews were guided by constructs from the Consolidated Framework for Implementation Research (CFIR), and qualitative comparative analysis (QCA) was used to uncover the configurations of CFIR factors that led to PrEP implementation.

**Results:**

We identified 3 distinct construct configurations, or pathways, that led to successful PrEP implementation: (1) high “Leadership Engagement” AND high “Available Resources”; OR (2) high “Leadership Engagement” AND NOT located in the Southeast region; OR (3) high “Access to Knowledge and Information” AND NOT located in the Southeast region. Additionally, there were 2 solution paths that led to absence of PrEP implementation: (1) low “Access to Knowledge and Information” AND low “Leadership Engagement”; OR (2) low “Available Resources” AND high “External Partnerships”.

**Discussion:**

We identified the most salient combinations of co-occurring organizational barriers or facilitators associated with PrEP implementation across Title X clinics in the Southern U.S. We discuss implementation strategies to promote pathways that led to implementation success, as well as strategies to overcome pathways to implementation failure. Notably, we identified regional differences in the pathways that led to PrEP implementation, with Southeastern clinics facing the most obstacles to implementation, specifically substantial resource constraints. Identifying implementation pathways is an important first step for packaging multiple implementation strategies that could be employed by state-level Title X grantees to help scale up PrEP.

**Supplementary Information:**

The online version contains supplementary material available at 10.1186/s43058-023-00450-2.

Contributions to the literature • Healthcare settings can exhibit significant heterogeneity in organizational context and barriers to implementation. Therefore, to promote intervention adoption, implementation strategies may need to be tailored to different organizational settings.

• Configurational comparative methods (CCMs), such as qualitative comparative analysis, are useful tools for implementation scientists to identify the multiple pathways or “recipes” that lead to implementation success.

• We demonstrate the application of CCMs to inform implementation strategy selection and planning for the scale-up of PrEP across a diverse network of family planning clinics.

## Introduction

In the U.S., women continue to face a significant burden of HIV, representing approximately 20% of new diagnoses [1]. Of these, nearly 60% occur among women in the Southern U.S. [1]. Compared to the national rate of newly diagnosed HIV infections (14 per 100,000 population), the rate in the Southern region (19 per 100,000), is significantly higher [2]. Pre-exposure prophylaxis for HIV (PrEP) is an effective and acceptable method of HIV prevention for women [3–6] when available, with options of daily oral tenofovir disoproxil fumarate/emtricitabine approved for cisgender women since 2012 (TDF/FTC) [4–6], or a cabotegravir bimonthly PrEP injection approved for cisgender women since late 2021 [7]. Both formulations of PrEP offer significant protection against HIV infection upon exposure when taken correctly [7], and oral PrEP has consistently been recommended for women at high risk of HIV through heterosexual contact since 2012 [8]. While recent years have seen more people accessing daily oral PrEP, women significantly lag behind men in uptake, representing fewer than 7% of U.S. PrEP users as of 2016 [1, 9, 10], and only 8% in 2021 [11]; as of 2019, it was estimated that only 10% of cisgender women with indication for PrEP had received a prescription [12].

Women face significant barriers to accessing PrEP, [13] most notably knowledge and availability of PrEP in settings where they routinely seek care. HIV prevention services often target men who have sex with men, [14, 15] while women’s health and family planning (FP) settings have yet to widely offer PrEP [14, 16–18]. Clinical settings are noted as acceptable settings to U.S. women for targeted HIV prevention [19, 20] and are a context in which upwards of 80% of women seeking services are at risk for unintended pregnancy [21], indicating heterosexual contact conducive to HIV transmission, the pathway responsible for 86% of HIV transmission among women [22, 23].

FP clinics have faced challenges implementing PrEP care, despite women’s perceived acceptability of being offered PrEP [24–27]. Identified barriers to PrEP implementation in FP clinics include resource availability (monetary, screening tools), [18, 28–31] lack of provider and staff knowledge, [23, 30, 32] competing priorities, [30] and negative provider attitudes towards PrEP [10]; training, [30] educational and screening resources, [30] leadership support, [10] and partnerships with local community organizations [30] act as facilitators. Addressing barriers, as well as reinforcing facilitators of PrEP implementation in FP clinics, must be prioritized to further equitable access to PrEP for women.

While numerous studies have assessed knowledge and attitudes among FP providers, [10, 18, 30, 31, 33, 34] fewer have employed the lens of implementation science to identify organizational-level facilitators and barriers to PrEP provision [10, 18, 31]. Almost entirely lacking are studies that identify singular or combinations of organizational factors that primarily drive or inhibit the facilitation of PrEP provision across FP clinics [31]. Honing in on these drivers of PrEP adoption and implementation across FP clinics can allow for the development and use of targeted implementation strategies that are most likely to facilitate PrEP implementation. As such, the purpose of this study is to use qualitative comparative analysis (QCA), a novel technique for assessing combinations of organizational factors contributing to an outcome of interest (i.e., provision of PrEP care), to hone the identification of implementation strategies to improve PrEP delivery in FP clinics across the Southern U.S.

## Methods

### Study design

The larger implementation study used an explanatory, sequential mixed-methods research design [35] to explore barriers/facilitators to PrEP provision in FP clinics across the U.S. South [including the Southeastern, Southwestern, and Mid-Atlantic regions of the U.S. with high levels of HIV and low levels of PrEP uptake]. Quantitative surveys administered online in Spring 2018 are described elsewhere [36, 37]; this study focuses on the results of subsequent qualitative interviews. Interviews were conducted from March to July 2018. This study protocol was approved by the Emory University Institutional Review Board (See Additional file 1 for STROBE checklist).

### Study participants and recruitment

We invited FP providers and clinic administrators from Title X-funded clinics in the 18 states that comprise the Southern U.S. (Department of Health and Human Services (DHHS) regions III [Mid-Atlantic], IV [Southeast], and VI [Southwest]) to participate. Title X clinics comprise the largest federally-funded sexual health network across the U.S. [38], with Title X funding allocated at the state level. Most states’ Title X funding is administered by a single grantee; the state-level grantee(s) distributes Title X funds to clinical service sites and is charged with building capacity of these clinical sites to provide comprehensive FP and other sexual health services (HIV testing/prevention, STI testing, Paps, etc.) through provision of training and technical assistance. FP providers were considered individuals who could prescribe, counsel, or screen for PrEP. Clinic administrators were individuals who served in an administrative oversight capacity over the Title X activities in their clinic.

Within the survey for the parent study, [36] participants indicated willingness to participate in a qualitative phone interview. To increase interview representativeness of FP clinics, individuals were purposively recruited based on clinic factors such as provision of PrEP, state, DHHS region, clinic classification (i.e., health department, community clinic, etc.), and urbanicity. Of the 519 survey participants representing 283 unique Title X clinics, 257 participants agreed to be contacted for a follow-up interview. In total, we interviewed 45 participants from 38 unique clinics; 7 clinics had 2 interviewees participate. The distribution of clinic characteristics (PrEP provision, region, clinic type and urbanicity) among the interviews mirrored the distribution observed among the survey participants [10]. Interviews took 45–60 min and participants received a $50 gift card upon completion.

### Measures

There are two outcomes for this QCA analysis: (1) presence of PrEP implementation and (2) absence of PrEP implementation in the clinic, based on participants’ reporting in the interview. Semi-structured interviews were guided by constructs from the 2009 version of the Consolidated Framework for Implementation Research (CFIR) [39] and adapted from interview questions provided on the CFIR website (www.cfirguide.org). The guide covered nearly all CFIR domains including Inner Setting factors (e.g., structural characteristics, culture, climate, resources, leadership, priorities, access to PrEP information, compatibility), Outer Setting factors (e.g., policies, external partnerships, patient needs, and peer pressures), Characteristics of Individuals (e.g., provider/staff PrEP attitudes), Intervention Characteristics (e.g., complexity, relative advantage), and Process (e.g., planning for adoption and engaging). Selection of constructs/questions included in the interview guide was based on prior literature on PrEP implementation [10, 31, 34, 40] and informed by discussions between experts with extensive prior experience working in these clinical environments and experience providing HIV and PrEP care to underserved communities in the South. Phone-based interviews were conducted by trained research staff; interviews were recorded and transcribed verbatim. Descriptive characteristics of the clinics (region, provision of primary care on-site, clinic type, urban/rural location, county-level poverty rate, county-level un-insurance rate, and county-level HIV rate) were extracted from the survey data and publicly available data.

### Data analysis

The overall analytic approach for this study was qualitative comparative analysis (QCA). In brief, QCA is an analytic technique for performing cross-case (i.e., cross-clinic) comparisons to identify conditions (or combinations of conditions) that are “sufficient” for an outcome of interest to occur [41–45]. Using Boolean algebra and minimization algorithms, QCA systematically compares cases and derives solutions consisting of one or more patterns of conditions that are necessary for the presence of an outcome (i.e., PrEP implementation) [41–45]. QCA is able to use relatively small data sets as there is no requirement to have enough cases to achieve statistical significance: a recent review found that the average number of cases used in QCA analyses is 20 (range: 5–50) [41]. In this study, we wanted to determine which combinations of CFIR constructs identified from the qualitative interviews and clinic characteristics (reported in the survey data or publicly available sources) are sufficient to lead to (1) PrEP implementation or (2) lack of PrEP implementation. It is common practice in QCA analyses to model both the presence and absence of an outcome, since pathways that facilitate implementation are often different from pathways that inhibit implementation [41]. Below we describe the multi-step process for conducting the QCA including (1) coding the qualitative data, (2) assigning ratings to each CFIR construct, (3) reducing and calibrating the data for analysis, (4) conducting the qualitative comparative analysis, and (5) describing qualitative case examples.

#### Qualitative coding

CFIR was the analytic framework for this study. We began by coding transcripts for each of the CFIR constructs, employing standard qualitative data analysis methods including reading of transcripts, creation of a codebook, coding, and consensus meetings [46]. To develop the codebook, CFIR constructs were operationalized in the context of PrEP implementation, with inclusion and exclusion criteria based on guidance provided on the CFIR website (www.cfirguide.org). Code definitions and criteria were iteratively adapted throughout the coding process. Independent coding of passages relevant to the CFIR domains and constructs was conducted by two analysts, followed by comparison of codes and resolution of all discrepancies through discussion. All coding was conducted in NVivo version 12.

Following qualitative coding, we narrowed down the number of CFIR constructs to include in subsequent analyses, based on findings from our qualitative coding, our extensive knowledge of the clinics (e.g., cases), and prior PrEP implementation literature [10, 31, 34, 40]. Although we coded for 17 CFIR constructs, only a limited number can be included in QCA, given the exponential increase in possible configurations with each additional construct and the increasing probability of getting a solution by chance [47]. QCA is well-suited for constructs that exhibit heterogeneity across organizations, and that have hypothesized connections to implementation success [41]. Therefore, we utilized results from our qualitative coding to eliminate constructs with minimal inter-clinic variation (e.g., complexity, culture, compatibility, priority, individual characteristics, policies), constructs infrequently discussed by participants (e.g., relative advantage, peer pressure), and constructs that participants did not endorse as contributing to PrEP implementation (e.g., structural characteristics, planning and engaging). CFIR constructs included in subsequent analyses included Available Resources, Implementation Climate, Access to Knowledge and Information, Leadership Engagement, Patient Needs and Resources, and External Partnerships. Our selection of these 6 constructs was also aligned with our knowledge of the clinics and findings from previous studies conducted in this setting [10, 31, 34, 40]. Construct definitions are provided in Table 1.

**Table 1:** Description of measures

**Variable name**	**Description**	**Raw data categories**	**Data reduction**^**a**^	**Included in QCA model iteration predicting implementation presence**	**Included in QCA model iteration predicting implementation absence**
Available resources	To what extent does the clinic have resources (e.g., money, staffing, time, and facilities) to implement PrEP?	1 = Very low 2 = Low 3 = Moderate 4 = High 5 = Very high	0 (low resources) = very low, low, moderate 1 (high resources) = high, very high	**Yes**	**Yes**
Implementation climate	To what extent do individuals in the clinic prioritize and value HIV prevention interventions such as PrEP?	1 = Very low 2 = Low 3 = Moderate 4 = High 5 = Very high	**_**	No	No
Access to knowledge and information	To what extent are staff and providers in the clinic trained/knowledgeable on HIV prevention and PrEP?	1 = Very low 2 = Low 3 = Moderate 4 = High 5 = Very high	0 (low access to knowledge) = very low, low, moderate, high 1 (high access to knowledge) = very high	**Yes**	**Yes**
Leadership engagement	To what extent does the clinic have leaders/champions who are willing to prioritize HIV services and advocate for PrEP implementation?	1 = Very low 2 = Low 3 = Moderate 4 = High 5 = Very high	0 (low leadership) = Very Low, Low, Moderate, high 1 (high leadership) = very High	**Yes**	**Yes**
Patient needs and resources	To what extent are patients in the clinic in need of PrEP services (i.e., to what extent do patients exhibit high risk sexual behavior or other conditions that would make them eligible for PrEP?)	1 = Very low 2 = Low 3 = Moderate 4 = High 5 = Very high	**_**	No	No
External partnerships	To what extent is the clinic networked with external HIV providers or PrEP-providing organizations?	1 = Very low 2 = Low 3 = Moderate 4 = High 5 = Very high	0 (low partnerships) = very low, low, moderate 1 (high partnerships) = high, very high	No	**Yes**
Region	Region where the clinic is located	1 = Mid-Atlantic 2 = Southeast 3 = Southwest	0 = Not Southeast 1 = Southeast	**Yes**	No
Primary care	Clinic provides primary care services in addition to family planning	0 = No 1 = Yes	**_**	No	No
Clinic Type	Type of clinic	1 = Stand-alone FP clinic 2 = Health department 3 = Hospital-based 4 = FQHC/community clinic	**_**	No	No
Urbanicity	Clinic’s geographic location	0 = Rural 1 = Urban	**_**	No	No
Poverty rate	Poverty rate in the clinic’s county	0 = lower 50^th^ percentile 1 = upper 50^th^ percentile	**_**	No	No
Uninsured rate	Uninsured rate in the clinic’s county	0 = lower 50^th^ percentile 1 = upper 50^th^ percentile	**_**	No	No
HIV Rate	HIV rate in the clinic’s county	0 = lower 50^th^ percentile 1 = upper 50^th^ percentile	**_**	No	No

^a^This column depicts how the raw data categories were dichotomized

#### Construct ratings

After coding and selecting appropriate CFIR constructs, we then assigned ratings to each construct (available resources, implementation climate, access to knowledge and information, leadership engagement, patient needs and resources, and external partnerships) by clinic. We adapted our rating rules based on guidance provided on the CFIR website (www.cfirguide.org). Ratings followed a 5-point Likert scale and indicated the perceived degree to which each construct was present in the clinic: 1 = very low, 2 = low, 3 = moderate, 4 = high, 5 = very high. For instance, clinics that were exceptionally well-funded and well-staffed for PrEP implementation would receive a score of 5 (very high) for the Available Resources construct. Clinics that had no training or informational materials on PrEP provision would receive a score of 1 (very low) for Access to Knowledge and Information. To assign ratings, three analysts individually read and rated coded segments for each CFIR construct. Since we conducted a clinic-level study, interview data from participants in the same clinic were assigned a single collective score based on all coded data from that clinic, to gain a more robust picture of clinic operations and PrEP procedures. Analysts then met to discuss and compare their ratings; over 90% of the analysts’ ratings were either in full agreement or only 1 point different. In instances where analysts disagreed, discrepancies were resolved through discussion. The inter-rater comparison matrix, showing agreements/disagreements between analysts, is shown in Additional file 2. The complete raw dataset, which includes the final construct ratings, is shown in Additional file 3.

#### Data reduction

After rating the qualitative data and constructing our raw data set (see Additional file 3), we further reduced and calibrated the variables prior to running the QCA model. This is an important step since QCA models should be limited to the most salient constructs and variable categories and factors should be carefully calibrated to produce accurate and meaningful results [47]. To reduce the raw dataset, we used the minimally sufficient conditions (msc) function in the “cna” package in R version 4.2.0. Using methods described previously [48–54], the msc function looks across the variables in our raw dataset (i.e., the 6 selected CFIR constructs and 7 clinic characteristics listed in Table 1) and the 38 cases (i.e., clinics) and produces a table (i.e., condition table) with all possible configurations (i.e., combinations) of variables. We considered all 1-factor, 2-factor, 3-factor, and 4-factor configurations. We utilized the Boolean output in the condition table as well as our background knowledge of the cases and theoretical/practice-based expertise to guide the selection of a smaller subset of factors to include in model iteration. Specifically, this process guided our understanding of which configurations of CFIR constructs/clinic characteristics and which variable categories (e.g., very low, low, moderate, high, very high) had the strongest apparent connections to the outcome (i.e., presence or absence of PrEP implementation).

For each configuration, the condition table provides a *consistency* score ((number of clinics with configuration AND outcome present)/(total number of clinics with configuration)), *coverage* score ((number of clinics with configuration AND outcome present)/(total number of clinics with the outcome)), and *complexity* value (i.e., number of discrete conditions in a configuration). The presence of high consistency and high coverage scores suggests a strong connection between the configuration and target outcome. We initially set the consistency threshold to 0.75 and ranked the configurations by coverage, to determine which configurations had the highest coverage for that consistency threshold. We then iteratively raised the consistency threshold (in 0.05 increments until we reached 1.0) and continued to assess which configurations had the highest coverage across each threshold. We prioritized the selection of high-coverage configurations (coverage > 0.50) and variables that consistently ranked highly across thresholds. We also prioritized configurations with lower *complexity,* since they were more theoretically interpretable and better aligned with our background and knowledge of the cases. Also, from an implementation science perspective, lower complexity configurations are more meaningful, since they can inform feasible approaches to promote intervention scale-up. We ran these configurational analyses for both outcomes: (1) presence of PrEP implementation and (2) absence of PrEP implementation.

Ultimately, our knowledge of the cases and the condition table helped us select variables that were most connected to the presence of PrEP implementation, which included: available resources, access to knowledge and information, leadership engagement, and region. Variables most connected to absence of PrEP implementation included: available resources, access to knowledge and information, leadership engagement, and external partnerships. We also utilized the condition table to make decisions about if/how to reduce variable categories (e.g., very low, low, moderate, high, very high). We decided to dichotomize the constructs because there were very clear thresholds (or cut points) for each variable. For instance, endorsing “very high” leadership engagement was linked to implementation presence while the other categories (e.g., high, moderate, low, very low) were linked to implementation absence. Therefore, this variable was dichotomized into low leadership engagement and high leadership engagement categories. Table 1 depicts how the variable categories were reduced (Column 4, “Data Reduction”) and which variables were included in the QCA model iteration (Columns 5 and 6). In addition, the final “reduced” data set, which was used to conduct the subsequent QCA analysis, is depicted in Additional file 4.

#### Qualitative comparative analysis

After data was reduced, we ran a crisp-set QCA using the QCAPro package in R version 4.2.0 [55]. QCA analyses were conducted for each outcome: (1) presence of PrEP implementation and (2) absence of PrEP implementation. We iteratively ran the QCA model, starting with a consistency threshold of 0.75 and iteratively raising the threshold in 0.05 increments until we reached 1.0. For the first outcome (presence of PrEP implementation), the same two models occurred at every consistency threshold:

- M1: (high resources*high leadership) + (high leadership* not southeast region) + (high access to knowledge * not southeast region) <  =  > PrEP implementation
- M2: (high resources*high leadership) + (high leadership* not southeast region) + (high access to knowledge * low leadership) <  =  > PrEP implementation

Both models had an overall consistency and coverage score of 1. The only difference between model 1 and model 2 is the last term (high access to knowledge * not southeast region versus high access to knowledge * low leadership). Because high access to knowledge * not southeast region covered a significantly higher proportion of cases compared to high access to knowledge * low leadership (64% versus 9%), we selected model 1 as our final solution. For our second outcome (absence of PrEP implementation), we followed the same process, and there was only one model that occurred at all consistency thresholds. Our final model for the second outcome was:

- M1: (low resources * high external partnerships) + (low access to knowledge *low leadership) <  =  > absence of PrEP implementation

#### Qualitative case examples

After identifying the QCA solution configurations associated with presence/lack of PrEP implementation, we returned to the qualitative transcripts for interpretation. For each identified solution configuration, we reviewed the transcripts and qualitative coding of clinics that fit that configuration to develop narrative case examples describing how each configuration functioned to promote/inhibit PrEP implementation. Representative quotes were selected to further elucidate our findings.

## Results

### Clinic characteristics

Of the 38 unique clinics represented in qualitative interviews, 11 (29%) clinics prescribed PrEP and 27 (71%) did not. See Table 2 for additional clinic characteristics.

**Table 2:** Clinic characteristics, by PrEP implementation status

	**Total** (*N* = 38) *n* (%)	**PrEP** (*N* = 11) *n* (%)	**Non-PrEP** (*N* = 27) *n* (%)
**Urbanicity**			
Urban	28 (74)	9 (82)	19 (70)
Rural	10 (36)	2 (18)	8 (30)
**Clinic type**			
Health department	22 (58)	4 (36)	18 (67)
Family planning	4 (11)	3 (27)	1 (4)
FQHC/community clinic	10 (26)	4 (36)	6 (22)
Hospital	2 (5)	0 (0)	2 (7)
**Region**^a^			
Mid-Atlantic	16 (42)	6 (55)	10 (37)
Southeast	14 (37)	2 (18)	12 (44)
Southwest	8 (21)	3 (27)	5 (19)
**Primary care**			
Yes	12 (32)	4 (36)	8 (30)
No	26 (68)	7 (64)	19 (70)
**Percent poverty**, mean (min, max)^**b**^	17 (6, 27)	17 (6,21)	17 (7, 27)
**Percent uninsured,** mean (min, max)^**b**^	11 (4, 21)	11 (4, 21)	11 (5, 18)
**HIV prevalence rate,** mean (min, max)^**b**^	669 (55, 2590)	696 (57, 2590)	658 (55, 2307)

^a^Department of Health and Human Services regions Mid-Atlantic (Washington D.C., Delaware, Maryland, Pennsylvania, Virginia, West Virginia), Southeast (Alabama, Florida, Georgia, Kentucky, Mississippi, North Carolina, South Carolina, Tennessee), and Southwest (Arkansas, Louisiana, New Mexico, Oklahoma, Texas)

^b^Based on AIDSVu 2015 data from clinic’s county

### Qualitative comparative analysis results

#### Solution configurations associated with presence of PrEP implementation

**Fig. 1:**
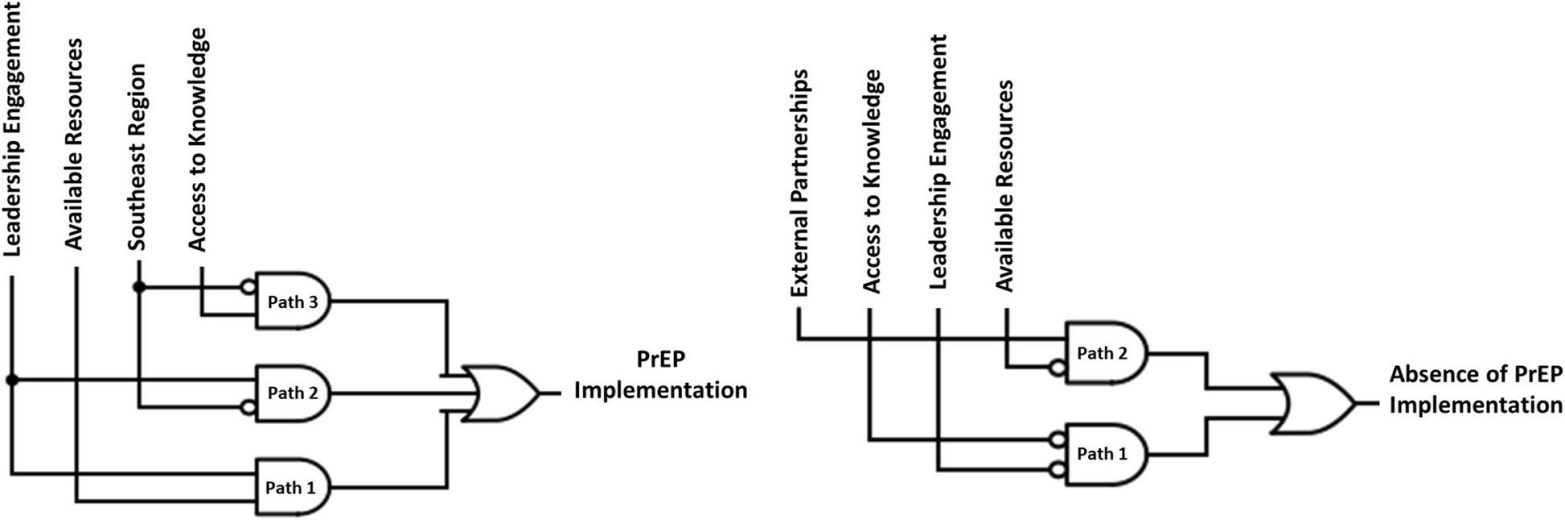
Solution pathways that led to implementation presence and absence. Solid lines indicate high levels of a condition and open circles indicate the low levels of a condition [56]

We identified 3 distinct solution paths that collectively explained 100% of PrEP-prescribing clinics with a consistency level of 100% (Table 3, Fig. 1). The presence of any of these 3 solution paths was sufficient for PrEP implementation to occur:

- Path 1: High leadership engagement AND high available resources (consistency = 100%, coverage = 82%) OR
- Path 2: High leadership engagement AND NOT located in the Southeast region (consistency = 100%, coverage = 73%) OR
- Path 3: High access to knowledge/information AND NOT located in the Southeast region (consistency = 100%, coverage = 64%)

**Table 3:**
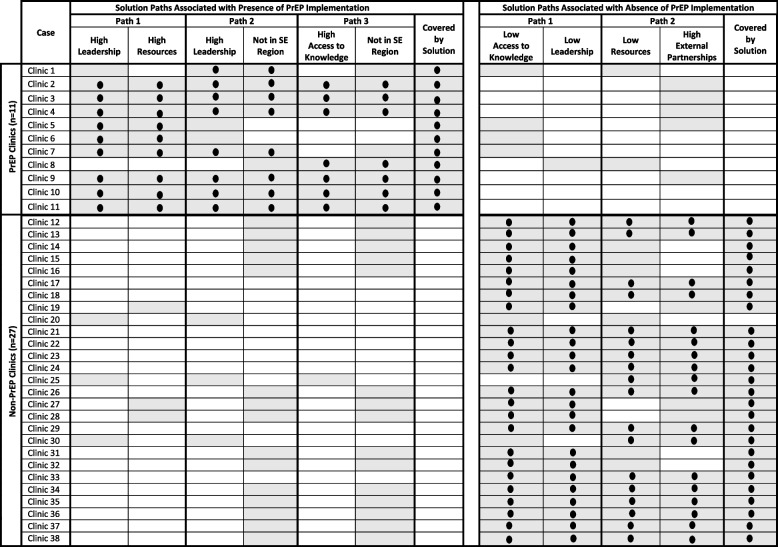
Matrix of all clinics and solution paths for implementation presence and absence

*Note*: Each row represents a different case (i.e., clinic) using de-identified site numbers. Shaded cells depict presence of conditions (e.g., leadership, resources, access to knowledge) and black dots represent cells covered by the solution

Each of the 3 solution paths was a conjunction of 2 factors, indicating that the presence of both factors is necessary for PrEP implementation. Path 1 occurred among 82% of PrEP clinics, Path 2 occurred among 73% of PrEP clinics, and Path 3 occurred among 64% of PrEP clinics. None of these solution paths occurred in the non-PrEP clinics (consistency = 100%). Table 3 shows a matrix display of all 38 family planning clinics and the solution paths. Six of the 11 PrEP-providing clinics had all three solution paths.

#### Solution configurations associated with absence of PrEP implementation

We identified 2 distinct solution paths that collectively explained 96% of non-PrEP clinics, with a consistency level of 100%. The presence of either path was related to a lack of PrEP implementation.

- Path 1: low access to knowledge/information AND low leadership engagement (consistency = 100%, coverage = 89%) OR
- Path 2: low resources AND high external partnerships (consistency = 100%, coverage = 70%)

Path 1 occurred among 89% of non-PrEP clinics and Path 2 occurred among 70% of non-PrEP clinics. None of these solution paths occurred in the PrEP-prescribing clinics (consistency = 100%). Of the 27 non-PrEP clinics, 16 clinics had both solution paths (Table 3).

### Qualitative case studies

#### Qualitative examples for presence of PrEP implementation

*Path 1*: Having clinic leaders who were committed to PrEP was vital to implementation. Specifically, clinics needed leaders who actively sought out resources and funding for PrEP implementation. Having committed leaders and available resources led to effective and efficient adoption among 82% of the PrEP-providing clinics:

But I do feel like in terms of an organization, the leadership has offered the education and the resources for us to offer it. Right now because the Department of Health has provided some funding for it, I think we're going to be able to reach another high-risk population, which are people using drugs. So I think that's going to put us in a good position to take an additional step towards HIV prevention using PrEP for patients that are interested. (Clinic 9)

*Path 2*: Among clinics that were not located in the southeast, high leadership engagement alone was enough to facilitate PrEP implementation. In these regions, there were incentives, guidance, and resources for PrEP implementation at the state level, so clinics did not need to plan for PrEP adoption as intensively as clinics in the southeast region. All that was needed was committed clinic leaders who wanted to utilize available guidance:

Our medical director is the one who basically is the one who makes all the final decisions as far as what we do in our clinic. But at the state level is where the PrEP program is really coming from and they’re the ones who have come down with all the paperwork and the forms and the guidelines and we basically follow what they have advised us to follow. I think it was decided through the state level that PrEP was going to be on the horizon. And our medical director was completely for it, completely on board. (Clinic 4)

*Path 3*: For other clinics not located in the southeast, access to knowledge and information alone was enough to facilitate PrEP implementation. For instance, having training on PrEP and having access to information in the clinic about PrEP was sufficient for implementation:

Our regional medical director recorded a PowerPoint presentation that went over PrEP. And then we also forward them any other information and fact sheets. We’ve received several different types of that from Truvada. Gilead had sent us some things. We forward those on to the managers at each clinic and the clinicians at each clinic. Yeah, that’s how we do a lot of the training. (Clinic 2)

#### Qualitative examples for absence of PrEP implementation

*Path 1*: Participants noted how leadership and training were intertwined, with leadership buy-in as vital to any consideration of implementing PrEP, while also being necessary for allocation of time for training. Participants believed that the lack of leadership engagement and training hindered their ability to adopt PrEP:

We’ve been trying [to get leadership] to just close the clinic down for a week … so we get all of our training. We are the health department, so we have lots of other trainings we have to do, that we have to do every single year … training that we are supposed to do on our own time. And to add another training, especially something as extensive as PrEP is hard. (Clinic 12)

*Path 2*: Some of these FP clinics, particularly those located within health departments, found that they did not have adequate resources (staff, funding, time, etc.) to implement PrEP, but also had fewer incentives to identify additional resources due to a high availability of PrEP services nearby (i.e., high external partnerships):

Truthfully I don’t know that it would be as feasible for us in our clinic. We do know … where the resources are and where we can refer folks. I know that sometimes like the funding and the expense of it might be an inhibitor. If we had someone that had questions about or we thought was a candidate, we know that we can refer them to a neighboring county to some different entities that do offer it. (Clinic 33)

## Discussion

Using QCA, our study has identified the most salient combinations of clinic characteristics and organizational factors associated with PrEP implementation or lack of implementation across 38 Title X-funded FP clinics across the Southern U.S. Our findings are aligned with those from the limited PrEP implementation literature among women’s healthcare settings, including our own prior studies specifically focused on FP clinics in the Southern U.S., identifying the availability of financial and staff resources, leadership engagement and access to knowledge and information about PrEP as key organizational-level factors associated with PrEP provision in these settings [10, 31, 34]. However, we extend this literature by identifying combinations of co-occurring organizational barriers or facilitators associated with PrEP implementation, which is a critical first step for packaging multiple implementation strategies to help scale up PrEP among Title X-funded clinics across this high HIV burden region.

According to Leeman and colleagues, scale-up implementation strategies are ideal for situations where there is an identified external agency charged with providing training and technical assistance to promote and support the adoption and implementation of new evidence-based practices within clinical sites [57]. Such is the case with Title X clinics, wherein the state-level grantee(s) manages and distributes Title X funds to clinics and supports capacity building for service provision through the provision of training and technical assistance. As such, our findings suggest that Title X state-level grantees in DHHS Regions III, IV, and VI, and possibly in other regions, should consider implementation strategies to enhance clinical capacity for PrEP implementation, including promoting clinic leadership engagement in PrEP implementation (e.g., leadership buy-in), access to PrEP information and training for clinic staff, and available resources (e.g., funding and staffing) that can be dedicated to PrEP implementation.

Regarding the QCA solution paths for PrEP implementation presence, the first path (high leadership engagement AND high available resources) is sufficient for PrEP implementation among 82% of the PrEP-providing clinics in our sample. This finding suggests that packaging strategies to enhance both leadership engagement and available resources may promote PrEP scale-up among a large proportion of DHHS Region III, IV, and VI Title X clinical network. Title X state-level grantees may consider packaging implementation strategies such as benchmarking and recognition systems to acknowledge success among clinical sites (to bolster leadership engagement) and learning collaboratives about PrEP provision among clinical sites (to learn strategies to overcome resource-related barriers common across Title X clinics). State grantees outside of DHHS regions III, IV, and VI should assess factors that may be most salient in their clinical contexts, to determine if such implementation strategy packaging may be effective more broadly.

Notably, path 1 (high leadership engagement AND high available resources) is the only identified pathway that is sufficient for PrEP implementation among clinics in the Southeast [DHHS region IV]. Clinics that are not in the Southeast were identified as having two additional pathways that can promote PrEP implementation: (1) high leadership engagement alone (see solution path 2) or (2) high access to knowledge and information alone (see solution path 3). This finding suggests that study clinics in the Mid-Atlantic [DHHS region III] and Southwest [DHHS region VI] regions have more pathways to PrEP adoption and that the additional pathways are qualitatively different from the pathway identified as applicable to all regions; therefore, the Mid-Atlanta and Southwest regions may have more and different options for packaging implementation strategies. Clinics in the Mid-Atlantic and Southwest also have pathways to implementation that rely on single factors (e.g., leadership engagement OR access to knowledge/information alone), suggesting that PrEP implementation may be simpler and more feasible in these regions. However, our findings indicate that clinics in the Southeast may have the most obstacles to implementation since they require two co-occurring factors (high leadership engagement AND high available resources) to promote implementation. These regional differences in PrEP provision may point to macro-level factors and resource constraints commonly reported as barriers to providing PrEP in Title X clinics in the Southeastern U.S. [10, 31, 34]. Specifically, all states in the Southeast have not expanded Medicaid, and none at the time of our study offered state-level PrEP assistance programs to offset costs associated with PrEP care, [58] both which could contribute to concerns about having the necessary resources to provide PrEP to low-income and/or uninsured patients – a sizeable portion of Title X clinics’ patient-population.  These regional differences were also supported by our qualitative narratives, where clinics in the Southeast consistently reported more barriers to PrEP adoption and specifically noted resource constraints (e.g., lack of staffing and funding) as key obstacles to PrEP adoption.

Somewhat encouragingly, Title X clinics matching our Path 2 solution for lack of PrEP implementation (i.e., low resources AND high external partnerships; this solution covered nearly all Southeastern clinics) indicated that despite resource constraints inhibiting their ability to provide PrEP, they felt they were connected to and could refer to other neighboring agencies that offered PrEP. Thus, for these clinics, and possibly those with similar contextual factors, the state-level Title X grantees could facilitate access to resources (e.g., training) to provide universal PrEP education to their patients, and provide support to strengthen linkages between Title X clinics and established PrEP programs to connect women to PrEP care.

Another pathway that led to a lack of PrEP implementation was low access to knowledge and information AND low leadership engagement. This pathway covered the majority (89%) of non-implementing clinics, suggesting that these co-occurring barriers are common among clinics not implementing PrEP. To help clinics with these co-occurring barriers implement PrEP, Title X grantees should consider packaging implementation strategies such as (1) training to build PrEP-specific capacity (to facilitate access to knowledge/information); (2) technical assistance and facilitation specific to PrEP (to facilitate access to knowledge/information); (3) PrEP implementation toolkits for use by Title X clinical sites (to facilitate access to knowledge/information); and (4) benchmarking and recognition systems to acknowledge success among clinical sites (to enhance leadership engagement).

### Limitations/strengths

This study was not designed to examine the impact of policies on service provision, so further research on macro-level influences on PrEP provision is needed to elucidate the driving factors behind the regional differences in PrEP provision we observed within Title X clinics across the Southern U.S. Additionally, QCA is only able to accommodate a small number of variables, which may limit the view of clinical barriers and facilitators to implementation. However, we utilized proven methods to identify factors that are most important for inclusion in the analysis. There is also the potential for measurement error and case misclassification, since the data was based on self-reported information, and loss of information due to variable response data reduction. Findings may not be generalizable beyond the contexts from which data was gathered- DHHS Regions III, IV, and VI- and further exploration of PrEP implementation in other clinical contexts is needed, particularly given the inability to control for potential confounders in QCA. It is possible that all potential solutions to PrEP implementation were not represented in our data, further underscoring the need for continued research; however, those solutions that were identified in this study are highly actionable and may facilitate PrEP implementation in the study contexts. Despite limitations, our mixed-methods study utilized QCA to uncover multiple combinations of organizational-level conditions that led to PrEP implementation success among a diverse sample of Title X clinics.

## Conclusions

The Title X family planning network is a vital source of sexual health care for nearly four million individuals across the U.S., including growing numbers of men. For many women, especially in states that did not expand Medicaid, Title X clinics serve as their sole source of health care. Our study identified the most salient combinations of co-occurring organizational barriers or facilitators associated with PrEP implementation across Title X clinics in the Southern U.S. This is an important first step for packaging multiple implementation strategies that could be employed by state-level Title X grantees to help scale up PrEP among Title X clinics in their states, and ultimately across this high HIV burden region.

## Supplementary Information

**Additional file 1.** STROBE Statement—Checklist of items that should be included in reports of cross-sectional studies.

**Additional file 2.** Inter-rater comparison matrix.

**Additional file 3.** Raw Dataset and Construct Ratings.

**Additional file 4.** Reduced Dataset for QCA Analysis.

## Data Availability

The datasets analyzed during the current study are available from the corresponding author on reasonable request.

## Abbreviations

CCMs: Configurational comparative methods
CFIR: Consolidated framework for implementation research
DHHS: Department of health and human services
FP: Family planning
HIV: Human immunodeficiency virus
MSC: Minimally sufficient conditions
U.S.: United States
PrEP: Pre-exposure prophylaxis
QCA: Qualitative comparative analysis
