# Supplementary material for: Using qualitative comparative analysis to understand the conditions that produce successful PrEP implementation in family planning clinics
Source: Implement Sci Commun. 2023 Jun 9;4:64. doi: 10.1186/s43058-023-00450-2 (PMC10251711; doi:10.1186/s43058-023-00450-2)
Supplement: Supplementary file 2 — Additional file 2. Inter-rater comparison matrix. [file 43058_2023_450_MOESM2_ESM.docx]

**Additional File 2. Inter-rater comparison matrix**

| **Clinic** | **PrEP** | **Available Resources** | | | **Implementation Climate** | | | **Access to Knowledge** | | | **Patient Needs** | | | **External Partnerships** | | | **Leadership Engagement** | | |
| --- | --- | --- | --- | --- | --- | --- | --- | --- | --- | --- | --- | --- | --- | --- | --- | --- | --- | --- | --- |
|  |  | Rater 1 | Rater 2 | final | Rater 1 | Rater 2 | final | Rater 1 | Rater 2 | final | Rater 1 | Rater 2 | final | Rater 1 | Rater 2 | final | Rater 1 | Rater 2 | final |
| Clinic 1 | Yes | 2 | 1 | 1 | 3 | 4 | 3 | 4 | 4 | 4 | 5 | 2 | 4 | 3 | 2 | 3 | 5 | 5 | 5 |
| Clinic 2 | Yes | 4 | 4 | 4 | 5 | 5 | 5 | 5 | 5 | 5 | 5 | 4 | 4 | 5 | 5 | 5 | 5 | 5 | 5 |
| Clinic 3 | Yes | 5 | 5 | 5 | 5 | 5 | 5 | 5 | 5 | 5 | 4 | 5 | 4 | 5 | 5 | 5 | 5 | 5 | 5 |
| Clinic 4 | Yes | 5 | 5 | 5 | 5 | 5 | 5 | 5 | 5 | 5 | 3 | 4 | 3 | 4 | 3 | 4 | 5 | 5 | 5 |
| Clinic 5 | Yes | 4 | 4 | 4 | 4 | 4 | 4 | 4 | 3 | 3 | 2 | 4 | 2 | 4 | 4 | 4 | 5 | 3 | 5 |
| Clinic 6 | Yes | 4 | 4 | 4 | 5 | 5 | 5 | 3 | 5 | 4 | 3 | 2 | 3 | 2 | 3 | 2 | 5 | 5 | 5 |
| Clinic 7 | Yes | 4 | 4 | 4 | 4 | 3 | 3 | 2 | 4 | 3 | 3 | 4 | 3 | 2 | 3 | 2 | 5 | 5 | 5 |
| Clinic 8 | Yes | 2 | 3 | 2 | 2 | 3 | 3 | 5 | 5 | 5 | 5 | 5 | 5 | 3 | 3 | 3 | 4 | 5 | 4 |
| Clinic 9 | Yes | 4 | 4 | 4 | 4 | 5 | 5 | 5 | 5 | 5 | 5 | 4 | 5 | 4 | 5 | 5 | 4 | 5 | 5 |
| Clinic 10 | Yes | 5 | 5 | 5 | 3 | 5 | 3 | 5 | 5 | 5 | 2 | 3 | 2 | 3 | 2 | 3 | 5 | 5 | 5 |
| Clinic 11 | Yes | 5 | 5 | 5 | 5 | 5 | 5 | 5 | 5 | 5 | 5 | 5 | 5 | 3 | 2 | 3 | 5 | 5 | 5 |
| Clinic 12 | No | 1 | 1 | 1 | 1 | 2 | 1 | 5 | 4 | 4 | 1 | 5 | 5 | 4 | 5 | 5 | 1 | 1 | 1 |
| Clinic 13 | No | 2 | 1 | 2 | 2 | 4 | 2 | 4 | 3 | 3 | 4 | 4 | 4 | 4 | 5 | 4 | 3 | 4 | 4 |
| Clinic 14 | No | 1 | 2 | 1 | 4 | 4 | 4 | 3 | 3 | 3 | 4 | 4 | 4 | 4 | 2 | 3 | 4 | 4 | 4 |
| Clinic 15 | No | 3 | 2 | 3 | 2 | 2 | 2 | 3 | 2 | 2 | 4 | 4 | 4 | 3 | 3 | 3 | 4 | 4 | 4 |
| Clinic 16 | No | 3 | 1 | 2 | 2 | 2 | 2 | 2 | 1 | 2 | 5 | 5 | 5 | 3 | 3 | 3 | 3 | 3 | 3 |
| Clinic 17 | No | 3 | 1 | 3 | 4 | 3 | 4 | 1 | 1 | 1 | 5 | 5 | 5 | 4 | 4 | 4 | 4 | 4 | 4 |
| Clinic 18 | No | 4 | 1 | 2 | 3 | 3 | 3 | 4 | 1 | 2 | 5 | 5 | 5 | 4 | 5 | 4 | 4 | 2 | 3 |
| Clinic 19 | No | 4 | 4 | 4 | 4 | 5 | 5 | 4 | 2 | 2 | 5 | 5 | 5 | 4 | 3 | 4 | 4 | 4 | 4 |
| Clinic 20 | No | 1 | 1 | 1 | 2 | 2 | 2 | 4 | 4 | 4 | 4 | 2 | 4 | 2 | 2 | 2 | 5 | 5 | 5 |
| Clinic 21 | No | 2 | 2 | 2 | 2 | 2 | 2 | 2 | 2 | 2 | 4 | 5 | 5 | 5 | 5 | 5 | 4 | 4 | 4 |
| Clinic 22 | No | 3 | 3 | 3 | 4 | 3 | 4 | 3 | 1 | 1 | 4 | 5 | 4 | 5 | 5 | 5 | 4 | 4 | 4 |
| Clinic 23 | No | 4 | 3 | 3 | 3 | 2 | 3 | 4 | 4 | 4 | 2 | 4 | 3 | 4 | 5 | 4 | 4 | 5 | 4 |
| Clinic 24 | No | 1 | 2 | 2 | 3 | 3 | 3 | 4 | 3 | 3 | 2 | 2 | 2 | 5 | 4 | 5 | 4 | 4 | 4 |
| Clinic 25 | No | 4 | 3 | 3 | 4 | 5 | 5 | 5 | 5 | 5 | 4 | 5 | 5 | 4 | 3 | 4 | 5 | 5 | 5 |
| Clinic 26 | No | 4 | 1 | 3 | 4 | 4 | 4 | 3 | 2 | 2 | 4 | 4 | 4 | 4 | 3 | 4 | 3 | 2 | 3 |
| Clinic 27 | No | 4 | 4 | 4 | 3 | 3 | 3 | 3 | 2 | 2 | 1 | 3 | 2 | 5 | 4 | 5 | 3 | 4 | 4 |
| Clinic 28 | No | 4 | 4 | 4 | 4 | 4 | 4 | 4 | 4 | 4 | 3 | 4 | 4 | 5 | 5 | 5 | 4 | 4 | 4 |
| Clinic 29 | No | 2 | 2 | 2 | 5 | 4 | 4 | 2 | 2 | 2 | 5 | 5 | 5 | 3 | 4 | 4 | 4 | 3 | 3 |
| Clinic 30 | No | 2 | 2 | 2 | 4 | 5 | 5 | 3 | 3 | 3 | 5 | 5 | 5 | 5 | 5 | 5 | 4 | 5 | 5 |
| Clinic 31 | No | 4 | 2 | 3 | 5 | 5 | 5 | 2 | 1 | 2 | 4 | 3 | 4 | 1 | 1 | 1 | 4 | 4 | 4 |
| Clinic 32 | No | 3 | 1 | 2 | 1 | 1 | 1 | 1 | 1 | 1 | 2 | 2 | 2 | 3 | 3 | 1 | 3 | 3 | 2 |
| Clinic 33 | No | 3 | 1 | 2 | 2 | 2 | 2 | 1 | 2 | 1 | 2 | 2 | 2 | 4 | 4 | 4 | 2 | 3 | 3 |
| Clinic 34 | No | 3 | 3 | 3 | 4 | 3 | 4 | 3 | 2 | 2 | 3 | 2 | 3 | 5 | 5 | 5 | 5 | 4 | 4 |
| Clinic 35 | No | 2 | 3 | 3 | 3 | 4 | 3 | 2 | 1 | 1 | 4 | 3 | 3 | 4 | 5 | 5 | 2 | 1 | 2 |
| Clinic 36 | No | 1 | 2 | 1 | 4 | 4 | 4 | 2 | 1 | 1 | 4 | 4 | 4 | 3 | 5 | 4 | 4 | 4 | 4 |
| Clinic 37 | No | 2 | 2 | 2 | 2 | 2 | 2 | 4 | 4 | 4 | 3 | 2 | 2 | 5 | 5 | 5 | 4 | 4 | 4 |
| Clinic 38 | No | 3 | 2 | 3 | 2 | 1 | 2 | 2 | 1 | 2 | 1 | 2 | 1 | 5 | 4 | 4 | 4 | 3 | 4 |

Notes: This table shows the individual scores for each rater (i.e., rater 1 and rater 2), and the final rating assigned to each construct based on discussion and consensus meeting between the two raters. Green shading indicates that the two raters completely agreed, yellow indicates the raters differed by one point, and red indicates that the raters differed by more than 1 point. Clinics above the dotted line prescribed PrEP and those below did not. 1= Very Low, 2= Low, 3= Moderate, 4= High, 5= Very High
