# Supplementary material for: Using qualitative comparative analysis to understand the conditions that produce successful PrEP implementation in family planning clinics
Source: Implement Sci Commun. 2023 Jun 9;4:64. doi: 10.1186/s43058-023-00450-2 (PMC10251711; doi:10.1186/s43058-023-00450-2)
Supplement: Supplementary file 3 — Additional file 3. Raw Dataset and Construct Ratings. [file 43058_2023_450_MOESM3_ESM.docx]

**Additional File 3. Raw Dataset and Construct Ratings**

| **Clinic ID** | **Prescribed PrEP ^a^** | **Available Resources ^b^** | **Climate ^b^** | **Access to Knowledge ^b^** | **Patient Needs ^b^** | **External Partnerships ^b^** | **Leadership Engagement ^b^** | **Region ^c^** | **Primary Care ^d^** | **Clinic Type ^e^** | **Urban ^f^** | **Poverty Rate ^g^** | **Uninsured Rate ^g^** | **HIV Rate ^g^** |
| --- | --- | --- | --- | --- | --- | --- | --- | --- | --- | --- | --- | --- | --- | --- |
| Clinic 1 | Yes | 1 | 3 | 4 | 4 | 3 | 5 | 3 | 0 | 2 | 0 | 1 | 1 | 0 |
| Clinic 2 | Yes | 4 | 5 | 5 | 4 | 5 | 5 | 3 | 0 | 1 | 1 | 1 | 1 | 0 |
| Clinic 3 | Yes | 5 | 5 | 5 | 4 | 5 | 5 | 3 | 1 | 4 | 1 | 0 | 1 | 1 |
| Clinic 4 | Yes | 5 | 5 | 5 | 3 | 4 | 5 | 1 | 0 | 2 | 1 | 1 | 0 | 0 |
| Clinic 5 | Yes | 4 | 4 | 3 | 2 | 4 | 5 | 2 | 0 | 1 | 1 | 0 | 1 | 1 |
| Clinic 6 | Yes | 4 | 5 | 4 | 3 | 2 | 5 | 2 | 0 | 1 | 1 | 1 | 1 | 1 |
| Clinic 7 | Yes | 4 | 3 | 3 | 3 | 2 | 5 | 1 | 0 | 2 | 0 | 1 | 1 | 1 |
| Clinic 8 | Yes | 2 | 3 | 5 | 5 | 3 | 4 | 1 | 1 | 4 | 1 | 0 | 0 | 1 |
| Clinic 9 | Yes | 4 | 5 | 5 | 5 | 5 | 5 | 1 | 1 | 4 | 1 | 0 | 0 | 1 |
| Clinic 10 | Yes | 5 | 3 | 5 | 2 | 3 | 5 | 1 | 0 | 2 | 1 | 0 | 0 | 0 |
| Clinic 11 | Yes | 5 | 5 | 5 | 5 | 3 | 5 | 1 | 1 | 4 | 1 | 0 | 0 | 0 |
| Clinic 12 | No | 1 | 1 | 4 | 5 | 5 | 1 | 3 | 0 | 2 | 1 | 1 | 1 | 0 |
| Clinic 13 | No | 2 | 2 | 3 | 4 | 4 | 4 | 3 | 0 | 2 | 0 | 1 | 1 | 0 |
| Clinic 14 | No | 1 | 4 | 3 | 4 | 3 | 4 | 3 | 0 | 2 | 0 | 1 | 1 | 0 |
| Clinic 15 | No | 3 | 2 | 2 | 4 | 3 | 4 | 3 | 0 | 2 | 1 | 0 | 1 | 0 |
| Clinic 16 | No | 2 | 2 | 2 | 5 | 3 | 3 | 3 | 0 | 2 | 1 | 1 | 0 | 1 |
| Clinic 17 | No | 3 | 4 | 1 | 5 | 4 | 4 | 2 | 0 | 2 | 1 | 1 | 1 | 1 |
| Clinic 18 | No | 2 | 3 | 2 | 5 | 4 | 3 | 2 | 0 | 2 | 1 | 1 | 0 | 1 |
| Clinic 19 | No | 4 | 5 | 2 | 5 | 4 | 4 | 2 | 0 | 2 | 0 | 1 | 0 | 0 |
| Clinic 20 | No | 1 | 2 | 4 | 4 | 2 | 5 | 2 | 0 | 1 | 0 | 1 | 1 | 0 |
| Clinic 21 | No | 2 | 2 | 2 | 5 | 5 | 4 | 2 | 1 | 4 | 1 | 0 | 1 | 1 |
| Clinic 22 | No | 3 | 4 | 1 | 4 | 5 | 4 | 2 | 1 | 4 | 1 | 0 | 1 | 1 |
| Clinic 23 | No | 3 | 3 | 4 | 3 | 4 | 4 | 2 | 1 | 4 | 1 | 0 | 1 | 1 |
| Clinic 24 | No | 2 | 3 | 3 | 2 | 5 | 4 | 2 | 1 | 3 | 1 | 0 | 1 | 1 |
| Clinic 25 | No | 3 | 5 | 5 | 5 | 4 | 5 | 2 | 1 | 4 | 1 | 1 | 1 | 1 |
| Clinic 26 | No | 3 | 4 | 2 | 4 | 4 | 3 | 1 | 0 | 2 | 1 | 1 | 0 | 0 |
| Clinic 27 | No | 4 | 3 | 2 | 2 | 5 | 4 | 1 | 0 | 4 | 1 | 0 | 0 | 0 |
| Clinic 28 | No | 4 | 4 | 4 | 4 | 5 | 4 | 1 | 1 | 2 | 0 | 0 | 0 | 0 |
| Clinic 29 | No | 2 | 4 | 2 | 5 | 4 | 3 | 2 | 0 | 2 | 1 | 0 | 0 | 1 |
| Clinic 30 | No | 2 | 5 | 3 | 5 | 5 | 5 | 2 | 0 | 2 | 1 | 0 | 1 | 1 |
| Clinic 31 | No | 3 | 5 | 2 | 4 | 1 | 4 | 1 | 0 | 2 | 0 | 0 | 0 | 0 |
| Clinic 32 | No | 2 | 1 | 1 | 2 | 1 | 2 | 1 | 1 | 4 | 0 | 1 | 0 | 0 |
| Clinic 33 | No | 2 | 2 | 1 | 2 | 4 | 3 | 2 | 1 | 2 | 1 | 1 | 1 | 0 |
| Clinic 34 | No | 3 | 4 | 2 | 3 | 5 | 4 | 1 | 0 | 2 | 1 | 1 | 0 | 1 |
| Clinic 35 | No | 3 | 3 | 1 | 3 | 5 | 2 | 1 | 0 | 2 | 1 | 1 | 0 | 1 |
| Clinic 36 | No | 1 | 4 | 1 | 4 | 4 | 4 | 1 | 0 | 2 | 0 | 0 | 0 | 1 |
| Clinic 37 | No | 2 | 2 | 4 | 2 | 5 | 4 | 1 | 0 | 2 | 1 | 0 | 0 | 0 |
| Clinic 38 | No | 3 | 2 | 2 | 1 | 4 | 4 | 1 | 0 | 3 | 1 | 0 | 0 | 0 |

Note: Clinics above dotted line implemented PrEP (e.g., implementation presence) and clinics below the line did not implement PrEP (e.g., implementation absence).

^a.^ Outcome Variable

^b.^ 1= Very Low, 2= Low, 3= Moderate, 4= High, 5= Very High. For instance, clinics that were exceptionally well-funded and well-staffed for PrEP implementation would receive a score of 5 (Very High) for the Available Resources construct

^c.^ 1= Mid-Atlantic, 2= Southeast, 3= Southwest

^d.^ 0= does not provide primary care, 1= provides primary care

^e.^ 1=Family Planning Clinic, 2=Health Dept, 3=Hospital, 4=FQHC/ Community Clinic

^f.^ 0= rural, 1= urban

^g.^ 0= lower 50^th^ percentile, 1= upper 50^th^ percentile
