# Supplementary material for: Using qualitative comparative analysis to understand the conditions that produce successful PrEP implementation in family planning clinics
Source: Implement Sci Commun. 2023 Jun 9;4:64. doi: 10.1186/s43058-023-00450-2 (PMC10251711; doi:10.1186/s43058-023-00450-2)
Supplement: Supplementary file 4 — Additional file 4. Reduced Dataset for QCA Analysis. [file 43058_2023_450_MOESM4_ESM.docx]

**Additional File 4. Reduced Dataset for QCA Analysis**

| **Clinic ID** | **Prescribed PrEP ^a^** | **Available Resources ^b^** | **Access to Knowledge ^b^** | **External Partnerships ^b^** | **Leadership Engagement ^b^** | **SE Region ^c^** |
| --- | --- | --- | --- | --- | --- | --- |
| Clinic 1 | Yes | 0 | 0 | 0 | 1 | 0 |
| Clinic 2 | Yes | 1 | 1 | 1 | 1 | 0 |
| Clinic 3 | Yes | 1 | 1 | 1 | 1 | 0 |
| Clinic 4 | Yes | 1 | 1 | 1 | 1 | 0 |
| Clinic 5 | Yes | 1 | 0 | 1 | 1 | 1 |
| Clinic 6 | Yes | 1 | 0 | 0 | 1 | 1 |
| Clinic 7 | Yes | 1 | 0 | 0 | 1 | 0 |
| Clinic 8 | Yes | 0 | 1 | 0 | 0 | 0 |
| Clinic 9 | Yes | 1 | 1 | 1 | 1 | 0 |
| Clinic 10 | Yes | 1 | 1 | 0 | 1 | 0 |
| Clinic 11 | Yes | 1 | 1 | 0 | 1 | 0 |
| Clinic 12 | No | 0 | 0 | 1 | 0 | 0 |
| Clinic 13 | No | 0 | 0 | 1 | 0 | 0 |
| Clinic 14 | No | 0 | 0 | 0 | 0 | 0 |
| Clinic 15 | No | 0 | 0 | 0 | 0 | 0 |
| Clinic 16 | No | 0 | 0 | 0 | 0 | 0 |
| Clinic 17 | No | 0 | 0 | 1 | 0 | 1 |
| Clinic 18 | No | 0 | 0 | 1 | 0 | 1 |
| Clinic 19 | No | 1 | 0 | 1 | 0 | 1 |
| Clinic 20 | No | 0 | 0 | 0 | 1 | 1 |
| Clinic 21 | No | 0 | 0 | 1 | 0 | 1 |
| Clinic 22 | No | 0 | 0 | 1 | 0 | 1 |
| Clinic 23 | No | 0 | 0 | 1 | 0 | 1 |
| Clinic 24 | No | 0 | 0 | 1 | 0 | 1 |
| Clinic 25 | No | 0 | 1 | 1 | 1 | 1 |
| Clinic 26 | No | 0 | 0 | 1 | 0 | 0 |
| Clinic 27 | No | 1 | 0 | 1 | 0 | 0 |
| Clinic 28 | No | 1 | 0 | 1 | 0 | 0 |
| Clinic 29 | No | 0 | 0 | 1 | 0 | 1 |
| Clinic 30 | No | 0 | 0 | 1 | 1 | 1 |
| Clinic 31 | No | 0 | 0 | 0 | 0 | 0 |
| Clinic 32 | No | 0 | 0 | 0 | 0 | 0 |
| Clinic 33 | No | 0 | 0 | 1 | 0 | 1 |
| Clinic 34 | No | 0 | 0 | 1 | 0 | 0 |
| Clinic 35 | No | 0 | 0 | 1 | 0 | 0 |
| Clinic 36 | No | 0 | 0 | 1 | 0 | 0 |
| Clinic 37 | No | 0 | 0 | 1 | 0 | 0 |
| Clinic 38 | No | 0 | 0 | 1 | 0 | 0 |

Note: Clinics above dotted line implemented PrEP (e.g., implementation presence) and clinics below the line did not implement PrEP (e.g., implementation absence).

^a.^ Outcome Variable

^b.^ 1= High, 0= Low

^c.^ 1= Southeast, 0= Not Southeast
